# Prediction of Outcome for Transabdominal Cerclage in Women with Cervical Insufficiency

**DOI:** 10.1155/2015/985764

**Published:** 2015-02-25

**Authors:** Ji Eun Song, Keun Young Lee, Ga Hyun Son

**Affiliations:** Division of Maternal Fetal Medicine, Department of Obstetrics and Gynecology, Hallym University College of Medicine, Seoul 150-950, Republic of Korea

## Abstract

We investigated pregnancy outcome following transabdominal cerclage (TAC) in women with cervical insufficiency (CI) and explored parameters for predicting pregnancy outcomes following TAC. In this retrospective cohort study, we included 161 women with TAC. We considered demographic, obstetric, and gynecologic histories, pre- and postoperative cervical length (CL), and CL at 20–24 weeks as parameters for predicting outcomes following TAC. Univariate and multivariate analyses were used to identify risk factors for predicting delivery before 34 weeks after TAC. 182 pregnancies occurred after TAC, and 290 pregnancies prior to TAC were identified. The rate of delivery <34 weeks significantly decreased following TAC (5% versus 82%, *P* < 0.001). Univariate analysis demonstrated that a short CL (<25 mm) at 20–24 weeks and adenomyosis were associated with delivery at <34 weeks' gestation following TAC (*P* = 0.015 and *P* = 0.005, resp.). However, multivariate analysis demonstrated that only a short CL (<25 mm) at 20–24 weeks was a significant predictor (*P* = 0.005). TAC is an efficacious procedure that prolongs pregnancy in women with CI. A short CL at 20–24 weeks may predict the delivery at <34 weeks' gestation following TAC.

## 1. Introduction

Cervical insufficiency (CI) accounts for 8% of fetal losses in the second trimester [1] and remains a major cause of neonatal morbidity and mortality. Conventionally, transvaginal cerclage (TVC) has been performed for CI. In 1965, Beson and Durfee devised transabdominal cerclage (TAC) for women who had unsuccessful pregnancies via TVC or in whom a TVC was not technically feasible [2]. Others have subsequently demonstrated highly improved perinatal outcome following TAC compared with the outcomes in their previous pregnancies [3–5].

Although TAC appears to be anatomically prudent, it is still reserved for selected women with CI, because TAC is more a surgically challenging procedure and is associated with higher morbidity rates than TVC [3–5]. Whereas reports on TAC outcomes have been published, the factors that predict the success of TAC have not been well established. Therefore, the objective of this study was to estimate the efficacy of TAC and to explore the factors that affect the success of TAC. We investigated whether demographic, obstetric, and gynecologic histories and other clinical factors such as cervical length (CL) and adenomyosis are important for predicting successful delivery after TAC.

## 2. Methods

This retrospective cohort study included data on singleton pregnancies in women with TAC between January 1999 and December 2009 at Hallym University Kangnam Sacred Heart Hospital. This study was approved by institutional review board of the hospital. The antenatal and delivery details were reviewed. The indications for TAC consisted of the following conditions: (1) extensively amputated cervix due to conization or trachelectomy, (2) congenitally short cervix, (3) marked scarring of the cervix after unsuccessful TVC, (4) severe multiple cervical defects due to obstetric trauma, (5) penetrating forniceal lacerations, (6) one or more previous TVC failures, and (7) one or more previous midtrimester losses after painless labor [2–5]. Maternal demographics, obstetric and gynecologic history, prior cervical surgery, TVC history, and uterine abnormalities were documented at the first visit. Failed TVC was defined as a vaginal cerclage that resulted in a nonviable pregnancy in women with CI. We excluded women who could not undergo TAC owing to genetic or structural fetal abnormalities, chorioamnionitis, PROM, or placental abruption and those with multiple pregnancies and chronic medical illness.

We estimated the outcomes of TAC compared with those at patients' prior pregnancies. Obstetric outcomes included gestational age at delivery, incidence of delivery <34 weeks of gestation, PROM, birth weight, and neonatal survival. Operative outcomes included intraoperative blood loss, intraoperative and postoperative complications such as rupture of uterine vessels, bladder and bowel injuries, abdominal pain, vaginal bleeding, cervical laceration, and erosion of the cerclage into the vagina. We also evaluated which factors among the maternal demographics, obstetric and gynecologic history, ultrasound assessment of CL, and adenomyosis were significantly associated with successful outcome of TAC.

TAC was performed by one surgeon (KYL). Under general anesthesia, initially, a Pfannenstiel incision was made through which the uterus was gently exteriorized. The cervicoisthmic region was exposed through sharp and blunt dissection of the vesicouterine peritoneum. The uterine vessels were displaced laterally to confirm avascular space. The avascular region was perforated with a right-angle clamp, and a 5 mm Mersilene tape (Ethicon, Somerville, New Jersey, USA) was passed through the tunnel from the anterior to posterior direction and was tied anteriorly. After assuring hemostasis, the uterus was placed back into the pelvic cavity, and the abdomen layers were closed, as done routinely.

All women underwent ultrasonography for the diagnosis of fetal abnormalities and adenomyosis, as well as underwent transvaginal ultrasonography of CL prior to TAC. Transvaginal ultrasonography has been reported to have high specificity and sensitivity in the diagnosis of adenomyosis and have high correlation with histologic diagnosis of adenomyosis [6, 7]. Adenomyosis was diagnosed when there were diffuse uterine enlargement, parallel shadowing, asymmetrical myometrial thickening, and ill-defined endometrial-myometrial junction [6, 7]. Women were routinely offered transvaginal ultrasound for measurements of CL postoperatively, and between 20 and 24 weeks following TAC. Transvaginal sonography was performed using the standard technique of a postvoid clear midsagittal view of the entire cervix [8]. All neonates were delivered by Cesarean section, and the TAC knot was left in situ for the possibility of future pregnancies, if the patient opted so at the time of the Cesarean section.

Student's *t* test was used for continuous variables and Pearson's *χ* ^2^ test or Fisher's exact test for categorical variables, where appropriate. McNemar's test was used to compare the likelihood of delivery <34 weeks of gestation before and after TAC. Univariate and multivariate analyses were used to examine significant predictors of the outcomes of TAC. A *P* value of <0.05 was considered statistically significant.

## 3. Results

We included 161 women who underwent TAC at a mean maternal age of 32 years (SD 3.2; range, 24–42 years). The mean gestational age at the time of TAC was 13.5 weeks (SD 0.8; range, 12–15). The maternal demographics are outlined in Table 1. Of 161 women, 100 (62%) had a previous failed vaginal cerclage and 32 (19.8%) had two successive failed vaginal cerclages. 75 (46.5%) had previously undergone cervical surgeries (conization or trachelectomy), which made TVC unfeasible. Two of 75 women underwent trachelectomy resulting from early stage cervical cancer.

There were no significant differences in preoperative and postoperative hemoglobin levels (11.7 ± 1.0 versus 11.3 ± 1.1 g/dL, *P* = 0.26). The median estimated blood loss was 100 mL (SD, 67; range, 50–1300 mL). Only four patients lost ≥500 mL due to uterine vessel rupture; however, no blood transfusions were required. No bowel or bladder injuries or cases of the TAC knot eroding into the vagina were reported. There was no cervical laceration or vaginal bleeding. There was a case of cramping abdominal pain and PROM within 24 hours of TAC; the miscarried fetus was delivered via dilatation and evacuation, leaving the cerclage in situ. This patient successfully carried a neonate to term one year later.

A total of 290 pregnancies prior to TAC were identified; a comparison of pregnancy outcomes before and after TAC is presented in Table 2. The mean gestational age at delivery prior to TAC was 23 weeks (SD, 3.6; range, 15–27), and the neonatal survival rate before TAC was 21% (61/290). Of the 161 women who underwent TAC, 21 had multiple subsequent pregnancies with an in situ TAC; therefore, the total number of pregnancies after TAC was 182. The mean gestational age at delivery after TAC was 36.3 weeks (SD, 4.9; range 15–39.8) with a neonatal survival rate of 96% (175/182). The mean birth weight was 2928 gram (SD, 806; range, 105–4580). PROM occurred in 8 cases; two occurred before 20 weeks and six occurred after 34 weeks. All neonates were delivered by Cesarean section after TAC. Two required Cesarean hysterectomy: one due to uterine atony and the other because of placenta previa totalis. Using McNemar's test, we compared the likelihood of delivery <34 weeks' gestation before and after TAC and found that after TAC, delivery <34 weeks' gestation decreased by 66.5% (95% CI 54.4–78.5).

The factors predicting outcome after TAC are presented in Table 3. The success of TAC was defined as prolonging pregnancy to over 34 weeks' gestation. The risk factor categories were defined accordingly: elderly gravida (maternal age ≥35 years), obesity (body mass index (BMI) >30 kg/m) [9], and a short CL <25 mm [8]. A univariate analysis was conducted to individually evaluate each risk factor as a predictor of TAC. When maternal age was considered as a two-level categorical variable, it was not a significant predictor of TAC (*P* = 0.13). There were no significant effects of obesity and prior TVC history on the outcome of TAC. Similar statistically nonsignificant conclusions were observed with parity, assisted conception, induced abortion history, and prior cervical surgeries. Preoperative and postoperative CL were not predictive of the pregnancy outcome of TAC (*P* = 0.054 and *P* = 0.745, resp.). However, short CL at 20–24 weeks was significantly associated with preterm delivery at <34 weeks following TAC (*P* = 0.015). Multivariate analysis controlled for maternal age, BMI, preoperative CL, and adenomyosis also showed that CL at 20–24 weeks was a significant predictor (*P* = 0.01, OR 9.96, 95% CI 1.71–58.01). Of note, univariate analysis revealed that maternal adenomyosis was correlated with preterm delivery at <34 weeks (*P* = 0.005). However, using multivariable analysis, adenomyosis was not a significant predictor of the outcome of TAC, with a *P* value of 0.20 (OR, 3.023, 95% CI 0.55–16.60).

## 4. Discussion

We found that TAC markedly improved perinatal outcome compared to those in their previous pregnancies before TAC; this finding is similar to those of previous studies [3–5]. TAC is an intervention not commonly performed in CI patients due to the operative challenge and higher morbidity compared to TVC. Therefore, the reports on the outcomes of TAC are limited compared to those of TVC. Previously published studies have found that women who undergo TAC have a survival rate between 60 and 96% [3–5]. The favorable outcome of TAC might be in part because this knot is tied more superiorly than in TVC, thus preventing a further inflammatory response prolonging the pregnancy. Our data also indicates a higher fetal survival rate (96%) and a higher rate of delivery at ≥34 weeks of gestation after TAC than that in their previous pregnancies without TAC (before TAC 18% versus after TAC 95%, *P* < 0.001). We further analyzed the likelihood of delivery at <34 weeks of gestation before and after TAC; our result suggests that the number of deliveries at <34 weeks of gestation prior to TAC was reduced by 66.5% after TAC (95% CI 54.4–78.5). In our study, there were no major complications such as massive hemorrhage at the time of TAC and PROM following TAC occurred in only eight cases (4%) (8/182), six of which ended in viable deliveries. Therefore, TAC is an efficacious and safe procedure when performed by a skilled surgeon.

Our results suggest that multiple potential risk factors can predict the outcome of TAC. Although successful outcomes of TAC have been reported, predictors of the success of TAC have not been thoroughly evaluated. As per previous studies, elderly gravida and assisted conception are well-known risk factors for preterm birth [10]. A recent meta-analysis concluded that obese women have a higher risk of preterm birth [11], and recent large epidemiologic studies have reported that induced abortions are a risk factor for preterm birth [12–14]. Further, a history of preterm birth is considered a strong indicator for future preterm birth [15]. Cervical surgeries and congenital uterine anomalies also are known to be a high risk for preterm delivery [16, 17]. However, our study suggested that the abovementioned factors were not significant contributors for predicting delivery <34 weeks of gestation in women with TAC.

The current study found that a CL >25 mm at 20–24 weeks of gestation was a predictor of success of TAC. CL has been shown to be a reproducible and reliable predictor of preterm birth. The efficacy of CL screening to prevent spontaneous preterm delivery has been investigated in women with a history of preterm deliveries [18–20]. Measuring the CL following TVC in the second trimester has been effective in predicting the outcome of pregnancy [21, 22]. However, the benefit of measuring CL in women with a TAC has not been thoroughly investigated in the literature. In our study, assessments of CL were performed preoperatively, postoperatively, and 20–24 weeks of gestation. Of these measurements, only CL at 20–24 weeks of gestation may help predict the success of TAC (*P* = 0.01). The shorter is the CL at 20–24 weeks of gestation, the higher is the incidence of preterm delivery at <34 weeks of gestation following TAC.

We demonstrated that a preoperative CL was not a predictor of preterm birth following TAC (*P* = 0.05). There was no association between postoperative CL and delivery at <34 weeks of gestation. In our study, the preoperative and postoperative CLs were measured approximately 14 weeks; the CL in the first trimester is usually ≥25 mm even in women at a high risk for preterm birth [18]. Our data on preoperative and postoperative CL are consistent with those of previous studies that demonstrated a CL before 14 weeks is not effective for predicting preterm delivery [18].

We found that maternal adenomyosis was a good predictor of TAC. However, further multivariate analysis revealed that adenomyosis was not a significant predictor of TAC (*P* = 0.20, OR 3.023, 95% CI 0.55–16.60). Pregnant women with adenomyosis are high risk for preterm birth [23], as it stimulates uterine contraction, which elevates intrauterine pressure and results in preterm delivery [24]. In our study, there were only 15 cases of adenomyosis. We postulated that the small number of patients with adenomyosis may not be sufficient to evaluate its effect as a predictor of the outcome of TAC. Therefore, the role of adenomyosis on the outcome of TAC warrants further studies. A larger sample size may allow us to obtain statistical significance for adenomyosis as a predictor.

The strength of our study is our simultaneous evaluation of multiple potential risk factors based on demographic, obstetric, and gynecologic histories and clinical factors. While our study is the first to assess the possible predictors of TAC, it is limited by its retrospective cohort study design, in which the cases serve as their own controls. Most published papers on the outcome of TAC use the patient's previous pregnancy as her own control [3–5], because it is difficult to provide appropriate matched controls. The most common indications for TAC are an extremely short cervix and prior failed TVC. In those with abnormally short or amputated cervix, TVC is technically impossible, which makes them unsuitable for comparison with TAC. There is one retrospective cohort study which compared TAC versus TVC in women with prior failed TVC [25]. In the study, patients with too short cervix for TVC placement were excluded [25]. Although the study provided matched controls, TAC in women with abnormally short cervix, which is one of the most common indications of TAC, was not evaluated. Unfortunately, there is no appropriate control population for patients with extremely short cervix. Our series included all classical indications for TAC including remarkably short cervix and prior failed TVC; therefore it was difficult to provide matched controls for comparison other than patient's own history. Using patient's own prior obstetric history as control has benefit of controlling for maternal characteristics that may act as confounding factors. Though a randomized controlled trial on TAC is warranted to assess more reliable benefits and risks of TAC, it would be practically difficult to perform the trial due to the difficulty in having sufficient numbers of TAC and finding appropriate control group. It will be more likely that patients with poor obstetric history may not agree to be randomized. Furthermore, it may be unethical to manage these patients expectantly who may benefit from TAC. An additional weakness of this study is that we could not determine biologic mechanisms explaining why CL at 20–24 weeks is a predictor of delivery <34 weeks of gestation following TAC. Therefore, subsequent research is needed to elucidate biologic mechanisms responsible for the favorable outcome of TAC.

## Figures and Tables

**Table 1:** Maternal characteristics.

Number of patients	161
Multiparity	129
Prior TVC in 1 or more pregnancies	100
Total number of TVC	125
Previous preterm delivery <34 weeks	131
Cervical surgery	75
Adenomyosis	15
Uterine anomaly	3
Induced abortion	
≥1 pregnancy	43
Total number of induced abortions	63
Alcohol use in pregnancy	0
Smoking	0

TVC: transvaginal cerclage.

**Table 2:** A comparison of pregnancy outcomes before and after TAC.

GA at delivery	Before TAC	After TAC	*P* value	95% CI
	*n* (%)	*n* (%)		
<34 weeks	239 (82%)	10 (5%)	<0.001	39.8–163.2
≥34 weeks	51 (18%)	172 (95%)	<0.001	

GA: gestational age; CI: confidence interval.

**Table 3:** Univariate analysis for the prediction of delivery before 34 weeks of gestation following TAC.

Independent variable	Univariate		
	OR	95% CI	*P* value
Maternal age (years)	0.210	0.027–1.645	0.137
BMI (kg/m^2^)	0.858	0.264–2.794	0.799
Multiparity	1.806	0.587–5.556	0.302
Assisted conception	1.891	0.613–5.833	0.267
Previous live birth history	1.200	0.400–3.602	0.745
Prior TVC history	2.478	0.770–7.977	0.128
Past preterm delivery	3.444	0.755–15.700	0.110
Induced abortion history	0.557	0.152–2.043	0.377
Previous cervical surgery	0.482	0.105–2.220	0.348
Adenomyosis	5.583	1.640–19.012	0.005^*^
Uterine anomaly	<0.001	<0.001–>999.999	0.986
Preoperative CL	4.196	0.974–18.065	0.054
Postoperative CL	1.015	0.928–1.111	0.745
CLat 20–24 weeks gestation	9.929	1.547–63.745	0.015^*^

OR: odds ratio; CI: confidence interval; BMI: body mass index; TVC: transvaginal cerclage; CL: cervical length.

^*^Statistically significant.
